# Efficacy of Porcine Epidemic Diarrhea Vaccines: A Systematic Review and Meta-Analysis

**DOI:** 10.3390/vaccines8040642

**Published:** 2020-11-02

**Authors:** Hokeun Won, Jeonggyo Lim, Yun Hee Noh, Injoong Yoon, Han Sang Yoo

**Affiliations:** 1 Department of Infectious Diseases, College of Veterinary Medicine, Seoul National University, Seoul 08826, Korea; hokeun@snu.ac.kr (H.W.); jason@cavac.co.kr (J.L.); 2 ChoongAng Vaccine Laboratories Co., Ltd., Daejeon 34055, Korea; sdocter@cavac.co.kr (Y.H.N.); iyoon@cavac.co.kr (I.Y.); 3 Bio-MAX/N-Bio Institute, Seoul National University, Seoul 08826, Korea

**Keywords:** systematic review, meta-analysis, porcine epidemic diarrhea (PED), vaccine efficacy

## Abstract

Porcine epidemic diarrhea (PED) is a devastating disease that causes considerable economic damage to the global pig industry. Although the causative agent, the porcine epidemic diarrhea virus (PEDV), was identified about a half century ago, there is still much debate on the preventive measures against the disease, especially regarding the PED vaccine. Recent reports on PEDV variants make the vaccination for PEDV more confusing. Therefore, we systematically reviewed published articles on PED and vaccines against the disease and performed a meta-analysis of vaccine efficacy based on the clinical signs, fecal score and survival rates. A total of 299 articles on the efficacy of PED vaccines were found online, and 21 articles were selected that fulfilled all the criteria. A meta-analysis was performed on the 21 articles based on the fecal scores and survival rates. This analysis showed the efficacy of PED vaccines, and no significant differences in the efficacy depending on vaccine type (killed vs. live) or administration route (intramuscular vs. oral) were found. The results from our study suggest that any vaccination against PED is a useful strategy to control the disease regardless of the type of vaccine and administration route.

## 1. Introduction

Porcine epidemic diarrhea virus (PEDV), a member of the genus *Alphacoronavirus* in the family *Coronaviridae* of the order *Nidovirales*, induces acute gastrointestinal symptoms characterized by dehydration, vomiting, diarrhea, and high mortality in newborn and suckling piglets [1]. PEDV was first identified in England and Belgium in the 1970s and has since been geographically restricted and problematic in Europe and Asia over the last three decades [2,3]. However, PEDV first emerged in the United States in 2013 and rapidly spread to adjacent North and South American countries, causing significant financial losses to their swine industries [4,5]. Then, the US prototype-like highly virulent G2b PEDV strains almost simultaneously invaded Asian countries, including South Korea, Taiwan, and Japan, resulting in the recurrence of a massive nationwide porcine epidemic diarrhea (PED) epidemic [6,7]. PEDV is now one of the most devastating porcine viruses that has emerged or re-emerged, presenting a significant threat to the worldwide pork industry [4,8,9].

G1a PEDV vaccines have been widely used in some Asian countries, including South Korea, China, Japan, and Thailand. Since 1999, three CV777-based inactivated and live-attenuated bivalent or trivalent vaccines have been used in China and a genotype 2a-based attenuated bivalent vaccine was introduced in 2015 [9]. The cell-adapted 83P-5 strain has been used as a live-attenuated vaccine (P-5V) in Japan [10]. The three cell-adapted PEDV strains, KPEDV-9, SM98, and DR-13 were used in Korea. The SM98 strain has been used intramuscularly as a live or killed vaccine, while DR-13 is available as a live oral vaccine [11,12,13]. However, some studies have raised questions about the efficacy of the vaccine since the highly virulent G2b PEDV emerged in the United States in 2013 and rapidly spread to its neighboring countries and Asian nations, causing considerable economic losses to their swine industries [4,9]. Owing to the prevalence of G2b PEDV throughout the world, some animal vaccine manufacturers and researchers are making efforts to develop G2b-based PEDV vaccines, considering the vaccine type (live or killed), the route of administration (intramuscular or oral), or antigen type (whole virus or recombinant protein). Accordingly, G2b whole-virus killed vaccines have been developed and used in the pig farms since 2014 in the US and 2015 in Korea, and a G2b live oral vaccine which was produced by Korean isolate, KNU-141113 S-DEL5/ORF3 strain has been approved in Korea and used in farms from 2020.

The process of systematic review has been accepted as a straightforward and replicable tool for synthesizing and analyzing the available data on the efficacy of interventions [14]. Meta-analysis is a research approach that statistically incorporates and objectively analyzes independent individual research findings on the same subject [15]. Meta-analysis has the advantage of increasing the number of research subjects by integrating the results of each research study into a weighted average summary calculation, increasing statistical power and precision, and overcoming the limitations of individual studies to obtain general, systematic and objective results [16].

A standard test model to evaluate the efficacy of the vaccine is crucial in developing an effective vaccine. However, there has not been a standard evaluation model for PED to date, and researchers typically use their own evaluation model based on previous studies. This research study is therefore intended to confirm the efficacy of the vaccine by systematically reviewing the efficacy evaluation model used in the PEDV vaccine studies published to date.

## 2. Materials and Methods 

### 2.1. Eligibility Criteria for Study

The criteria for the key question of this study were specified by the PICO (Population, Intervention, Comparison, Outcome) standard [14]. The population was the pigs that were administered PEDV vaccine. Intervention included the studies that conducted the efficacy test through challenge with a virulent PEDV after vaccination. For comparison, an unvaccinated control group was used. For outcome, fecal consistency score and survival rate after challenge were used.

### 2.2. Literature Search

According to mutual agreement on the inclusion criteria, the literature was searched using electric databases by two researchers. The literature was searched for studies investigating the effect of vaccines against PED on 30 April in 2020. The studies were written in either English or Korean, and they were identified using PubMed (http://www.ncbi.nlm.nih.org/pubmed/) and Web of Science (http://apps.webofknowledge.com/) for English studies and Research Information Sharing Service (RISS, http://www.riss.kr/index.do) for literature written in Korean. Medical subject headings (MeSH) were used to increase the sensitivity and specificity of the search. The keywords used for the search string in the database in English and translated into Korean are as follows:

“Porcine epidemic diarrhea” AND vaccine

All identified studies were reviewed to obtain information on PEDV vaccine type, vaccination age (i.e., sow, piglet), vaccination route (i.e., intramuscularly, per oral), and efficacy measures. We contacted the corresponding authors to obtain the raw data if we could not obtain the data even though the study met all eligibility criteria. In this meta-analysis, the term “study” was used to define published research, and the term “trial” was used to define the target animal testing with a challenge test conducted within a study.

### 2.3. Data Extraction

The fecal status after challenge was scored on a 4-point scale from 0 to 3 (0; normal and no diarrhea, 1; mild and fluidic feces, 2; moderate watery diarrhea, 3; severe watery and projectile diarrhea) in 10 of the 12 trials, and a score of 4 given in some papers was irrelevant to scoring fecal status as it was given when pigs died. One of the other two papers classified it into 3 stages [17], and the other into 6 stages from 1 to 6 points [18]. In most papers, the fecal score data were presented up until 1 to 2 weeks after challenge, but in some papers, they were presented until Day 5 or 21 after challenge. Since the most severe diarrhea was usually observed on Day 3 after PED challenge and most papers provided data for Days 3 to 6 after challenge, the data on Day 5 after challenge were collected and analyzed. If there were no data on Day 5, the data on the nearest date were used instead. Because the conditions of challenge (pig age on challenge day, type and pathogenicity of the challenge virus, virus dose, etc.) were different, it was deemed that collecting data on the same date for all studies was not truly meaningful. On the other hand, data on the survival rate at the end date of the experiment presented in each paper were used for the analysis, as was the value, although the observation period varied from 4 to 21 days.

### 2.4. Statistical Analysis

Meta-analysis models are divided into the fixed effects model, which assumes that the effect size for all groups is identical, and the random effects model, which assumes that the effect size of the population varies by study. In this study, the difference in effect sizes was investigated using both models, and the effect size was converted into Hedges’ g for interpretation. In general, the effect size is classified into small effect size (<0.3), medium effect size (0.3 to 0.8), and large effect size (≥0.8).

The effect size can vary among different studies, which is called heterogeneity, and a heterogeneity test was performed to check for heterogeneity. The I^2^ statistic, which evaluates the degree of heterogeneity, was used along with Q values. Generally, heterogeneity is low for I^2^ values of ≤25%, moderate for up to 50%, and high for up to 75% [14,19].

We analyzed the potential publication bias of the target study using a funnel plot [20]. A funnel plot is a tool used to present the likelihood of error, not to prove error, and the *X*-axis and the *Y*-axis display effect size and standard error, respectively. In general, studies with large sample sizes show a concentrated distribution around the mean at the top of the graph, while studies with small sample sizes show a relatively dispersed distribution at the bottom of the graph due to their large standard error [21]. When there was asymmetry in the funnel plot, Egger’s regression test was performed to calculate the exact figure, and a further analysis was performed using the trim-and-fill method to correct the asymmetry and estimate any change in the adjusted overall effect [22].

All calculations and analyses of this study were performed using Comprehensive Meta-Analysis Software version 2.0 (Biostat Inc., Englewood, NJ, USA).

## 3. Results

### 3.1. Systematic Analysis of Articles Related to PED

Three hundred and one studies were found after searching for “porcine epidemic diarrhea” and “vaccine” in PubMed and RISS. Two hundred and ninety-nine studies remained after excluding two duplicates. Of the 299 publications identified, 266 studies were excluded for the following reasons: 11 articles involved non-PEDV research, 185 publications were not for PEDV vaccinations, 19 studies used mice for testing, 30 papers performed pig experiments without a challenge test, and the remaining 21 articles were review papers. Therefore, 33 studies were selected in the first screening process, and these are summarized in Table 1. Of these, 10 studies were about live vaccines, and the other 23 studies were on killed vaccines. In 10 studies on live vaccines, the percentages of vaccines for oral and intramuscular injection were 80% and 20%, respectively. On the other hand, the percentages of vaccines for intramuscular, oral, and nasal injection in the studies on killed vaccines were 74%, 17%, and 9%, respectively. In addition, of those 33 studies, 16 evaluated vaccine efficacy by challenging piglets born from vaccinated sows, and the other 17 evaluated vaccine efficacy by challenging piglets after vaccination. In most of the studies, vaccine efficacy was evaluated through clinical observations and serological tests after PEDV vaccination and a challenge test. Some studies have even presented cellular immune responses or histological findings to identify vaccine efficacy. For the clinical observation results, the fecal consistency score, viral shedding, and mortality were individually provided in 91%, 85% and 52% of the studies. For the serological test, 94%, 73% and 67% of the studies showed the antibody titer of virus neutralizing (VN), IgG and IgA ELISA, respectively. All 33 studies analyzed the antibody titer by using at least one serological test (VN test or ELISA). Fifty-five percent of the trials had all three kinds of serological test results. Serum, colostrum, milk or whey was used for the VN test, and serum, colostrum or milk was used for the IgG ELISA. For the IgA ELISA, serum, colostrum, milk, whey, feces, saliva or mucous extracts was used.

**Figure 1:**
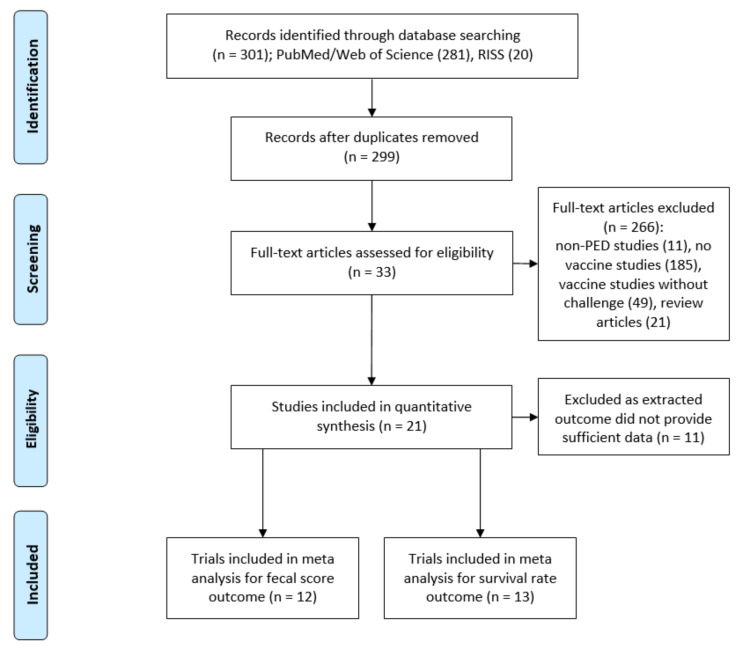
Flow chart of the article selection of the study (PRISMA flow diagram).

In our study, clinical observation data were analyzed to evaluate PEDV vaccine efficacy. Among the various evaluation indexes, survival rate and fecal consistency score after challenge, which have the clearest criteria, were used to perform a meta-analysis. Since numerous researchers performed their tests with different samples and various test methods in different periods of time, the serological test results were not appropriate for use in the meta-analysis. Therefore, 21 studies, which had data including number of animals, mean values, and standard deviations for the meta-analysis, were finally selected. The studies involved 12 trials with analysis of fecal scores and 13 trials with analysis of survival rates (Figure 1).

### 3.2. Meta-Analysis

#### 3.2.1. Effect of PEDV Vaccine

A meta-analysis was performed to determine whether there was a difference in the effect of the PEDV vaccine. The effect sizes of the fixed effect model and random effect model were 1.907 (Z = 11.775, *p* < 0.05) and 2.421 (Z = 6.028, *p* < 0.05), respectively, based on the fecal scores, which indicate statistically significantly large effect sizes. In addition, the effect sizes of the fixed effect model and random effect model based on the survival rates were 3.474 (Z = 9.109, *p* < 0.05) and 3.700 (Z = 8.138, *p* < 0.05), respectively, which also shows statistically significant large effect sizes (Table 2).

#### 3.2.2. Heterogeneity Assessment

**Figure 2:**
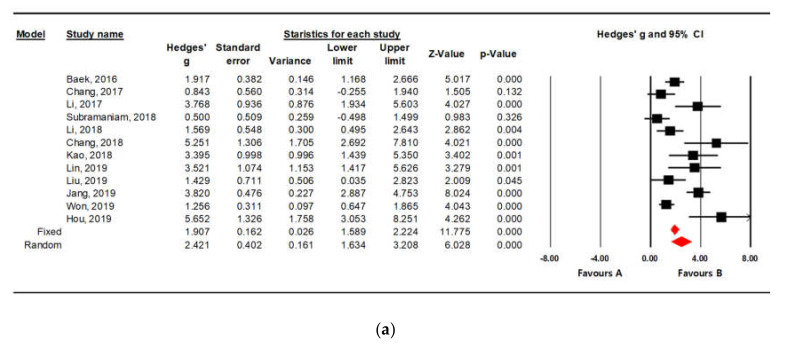

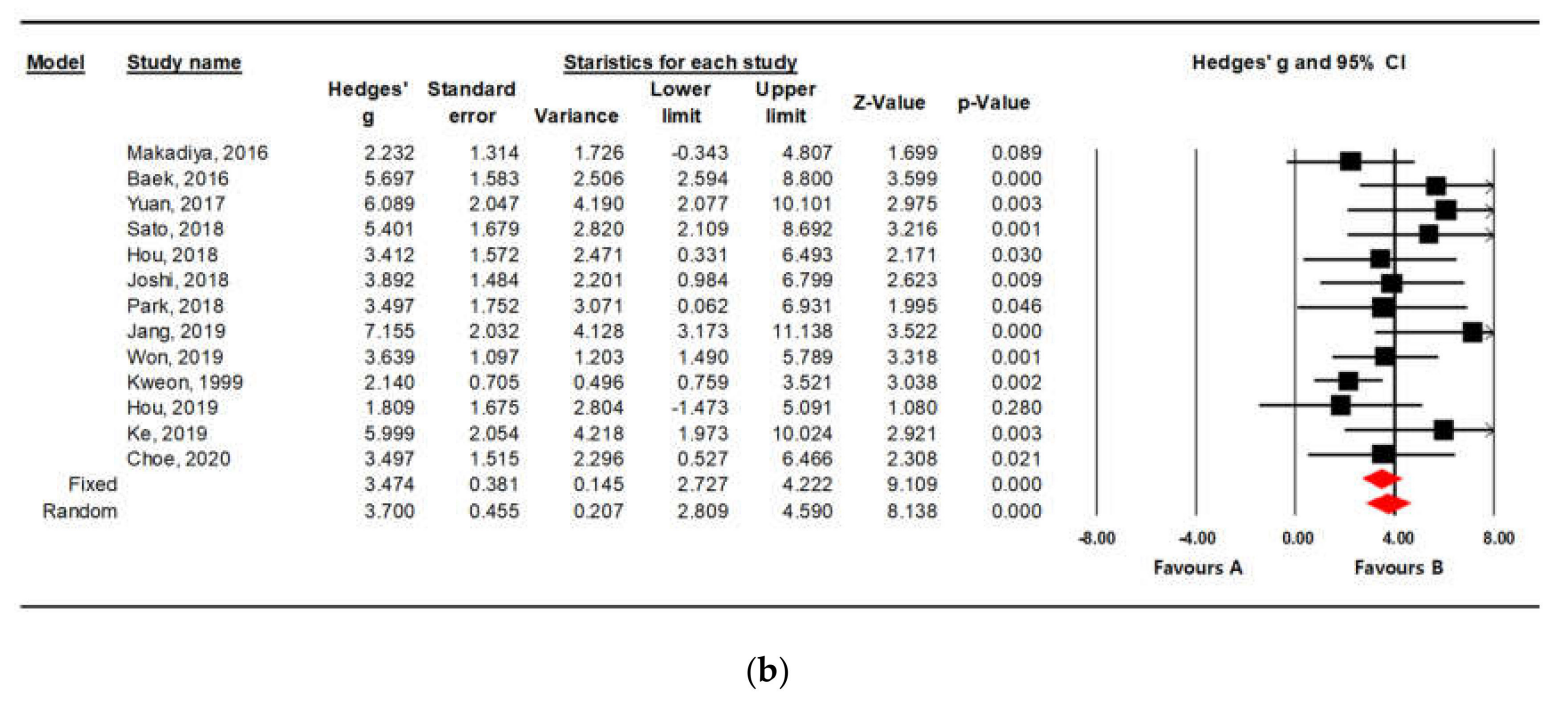
Forest plot for PEDV vaccine efficacy based on fecal score (**a**) and survival rate (**b**). Hedges’ g value indicates an effect size. Diamond at the bottom of the plot represents the average effect size of the studies.

The forest plots were visually consistent in orientation, but a heterogeneity test was performed for accurate evaluation. The analysis based on the fecal scores after challenge showed that the Q-value was 55.586 and the *p*-value was 0.000, which rejected the null hypothesis. Thus, the presence of heterogeneity was confirmed. To more specifically identify the degree of confirmed heterogeneity, the I^2^ statistic value was assessed. The I^2^ value was 80.211, so it can be concluded that there is severe statistical heterogeneity, which may indicate that various backgrounds of each study have an effect on the study results (Table 3). Since it was confirmed that there was heterogeneity in this study, the effect size was identified using the random effect model, and the effect size was 2.421 (Z = 6.028, *p* < 0.05) (Figure 2a).

On the other hand, when the efficacy of the PEDV vaccine was analyzed based on the survival rates, the Q-value was 15.286, and the probability value was 0.226. Thus, as the null hypothesis was not rejected, no heterogeneity was present. The I^2^ value, which identifies the degree of heterogeneity, was 21.498, which also indicated low statistical heterogeneity (Table 3). Thus, the effect size was determined by the fixed effect model. As a result, the effect size was 3.474 (Z = 9.109, *p* < 0.05) (Figure 2b).

#### 3.2.3. Publication Bias Analysis

The publication bias was verified through a funnel plot analysis that visually showed the error. The funnel plot was confirmed to be asymmetric for all studies based on fecal scores and survival rates, which suggested the existence of publication bias. To identify the exact value, Egger’s regression test was performed. The results of the fecal score and survival rate analyses showed probability values of 0.004 and 0.003, respectively. Thus, as the null hypothesis was rejected, publication bias was confirmed (Table 4). In other words, since the funnel plot was visually asymmetrical and there is a statistically significant relationship between effect size and standard error, based on the analysis result of Egger’s regression test, it can be said that sample size and effect size have a statistically significant relationship.

In addition, a classic fail-safe N was performed to identify how many research papers need to be added to change the final analyzed result of the meta-analysis. It was found that 480 and 297 additional studies were required for studies based on fecal scores and survival rates, respectively, to make the overall effect nonsignificant.

**Figure 3:**
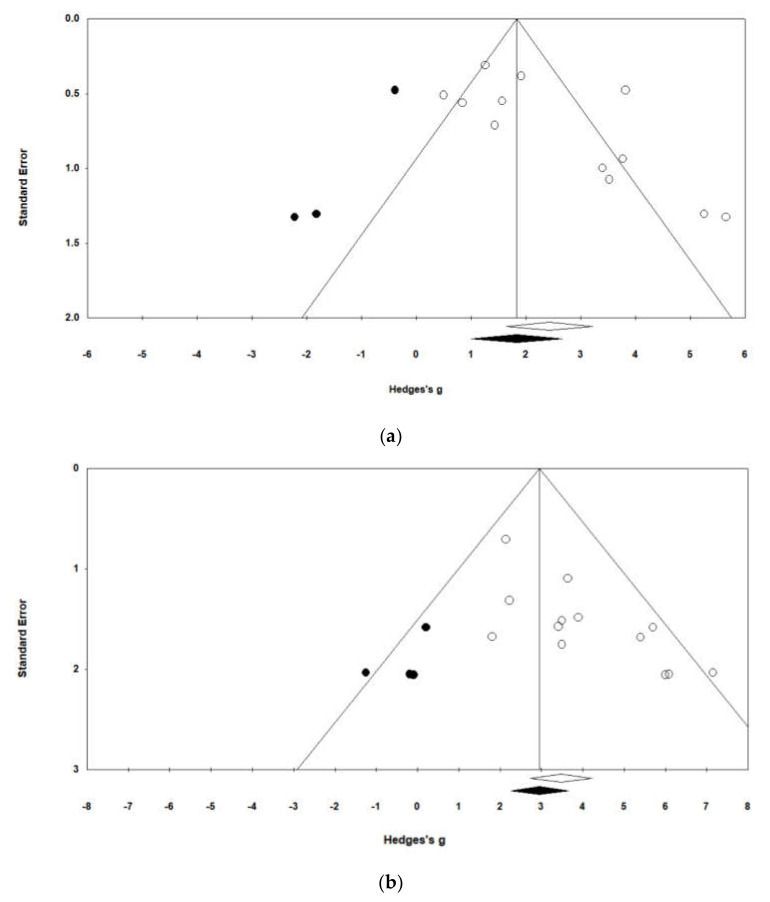
Funnel plot for publication bias from the studies on PEDV vaccine efficacy based on fecal score (**a**) and survival rate (**b**). The *X*-axis and the *Y*-axis of the funnel plot display effect size and standard error, respectively. The dark spots are the potential missing studies according to the trim-and-fill method.

As publication errors were shown in the overall effect size, we analyzed the effects of automatically missing data when the asymmetric funnel plot was made symmetric using the trim-and-fill method. According to the results of the fecal score analysis, three effect sizes not reported due to publication error were added (black dots), and the effect size of the random effect model changed from 2.420 to 1.828 (Figure 3a). The results of the survival rate analysis showed that four effect sizes that were not reported due to publication error were also added, and the effect size of the fixed effect model changed from 3.474 to 2.953 (Figure 3b). In other words, it can be said that both analyses have automatically corrected effect sizes considering the likelihood of publication error.

#### 3.2.4. Effect Analysis Based on PEDV Vaccine Type and Vaccination Route

##### Effect Analysis Based on PEDV Vaccine Type

The efficacy results of the PEDV vaccines used in this analysis were classified into killed vaccines and live vaccines, and the mean effect size of the two groups was compared to determine whether there was a difference in efficacy between the two vaccines. Based on the analysis of the fecal score data, the effect size of the killed vaccine was 1.675 (standard deviation: 0.424) and that of the live vaccine was 3.129 (standard deviation: 0.683). Analysis of the survival rate data showed that the effect sizes of the killed vaccine and the live vaccine were 4.109 and 3.173, respectively, with standard deviations of 0.672 and 0.463, respectively. In both groups, the effect size was significant, as their 95% confidence intervals do not include 0. The Q-value of the two groups was 3.270 (*p*-value: 0.071) based on the analysis of the fecal scores and 1.317 (*p*-value 0.251) based on the analysis of the survival rates. Thus, the effect size was confirmed to be the same between the groups. In other words, there is no statistically significant difference in the efficacy of PEDV vaccines between the killed vaccine and live vaccine (Table 5).

##### Effect Analysis Based on Vaccination Route

Additionally, the vaccination routes of PEDV vaccines were classified into intramuscular (IM) and oral (PO) vaccines, and the difference in the mean effect size was compared to determine whether there was a difference in the efficacy of the vaccine between the two groups.

As with the effect analysis based on the vaccine type, the same statistically significant effect size was recognized between the two groups. Therefore, no statistically significant difference in the efficacy of the vaccine based on the vaccination route was found (Table 6).

## 4. Discussion

Effective vaccines against recently prevalent G2b PEDV are actively under development around the world. However, since there is no definite target animal testing model to prove the efficacy of the vaccines, researchers have been conducting research on vaccine development by setting their own criteria for various efficacy assessments, such as fecal consistency and clinical symptoms, virus output, survival rates, and PED antibodies (IgA, IgG, and VN) in serum or colostrum after challenge. Thus, a meta-analysis was performed using papers published to date to determine whether PEDV vaccines are effective and which vaccine (live or killed vaccine and PO or IM) is more effective in preventing PEDV infection based on the various assessment items used in studies on PEDV vaccine development reported to date. A meta-analysis can generalize a number of existing studies that individually reported the effectiveness of the study through quantitative integration and is useful in that it provides a reasonable basis for clinical decision making [52].

For the literature search, “porcine epidemic diarrhea” and “vaccine” were set as the keywords, and English papers were searched using PubMed and Korean papers using RISS. As a result, 281 and 20 papers were found, respectively. After studies unrelated to a vaccine test, review articles, and papers where the vaccine test was conducted in pigs but a challenge was not performed were excluded, 21 papers were finally selected for this meta-analysis.

In the selected papers, the efficacy of PEDV vaccines was investigated by scoring fecal status and comparing survival rates and virus output with nonvaccinated control groups after challenge or by measuring IgA, IgG, neutralizing antibodies, etc., in the serum or colostrum of pigs. However, the analysis of real-time PCR data on the dose of virus for challenge excreted in feces or serum test results, which were used as a common evaluation index in many research papers on PEDV vaccines, could not be used in this meta-analysis since the numbers in the studies were too different to integrate the results of each study and there was a limit to collecting the information needed for the meta-analysis. In this study, the efficacy of PEDV vaccines was analyzed using data on fecal scores and survival rates of pigs after challenge.

In 10 out of the 13 trials that identified the survival rates of pigs after challenge, sows were vaccinated, and challenge was performed on their piglets. In two trials, 1-day-old and 4-day-old piglets were vaccinated and challenged. In a paper published by Yuan X et al. [44], 4-week-old minipigs were vaccinated, and they were challenged. Observation of the survival rates of the pigs after challenge was monitored for at least 4 days and up to 21 days depending on the paper. In this meta-analysis, survival rate analysis was performed using the final survival rate results presented after the end of the survival rate observation period for each study.

The results of the heterogeneity analysis confirmed that there was severe heterogeneity when using the fecal scores for analysis. The reasons for the severe heterogeneity were speculated to be due to different time intervals of challenge after vaccination, different ages of pigs on the challenge day, different pathogenicity of the challenge virus, and various concentrations and doses of the virus for challenge. In contrast, there was low heterogeneity when it was analyzed using the survival rate data after challenge. It was thought that the difference in the results of each study was relatively small because each study identified the dose of challenge virus that could induce death of pigs in the control group that were not vaccinated before the trial and then perform challenge relative to the virus dose. In fact, among the 13 trials that analyzed the survival rate of pigs after challenge, the survival rate of the control group was 0% to 20% in 10 trials, and in the other 3 studies, the survival rate of the control group was 42.9% to 66.7%.

An analysis of publication bias was conducted on the papers analyzed in this study. Publication bias refers to the bias where the results of a meta-analysis are distorted because positive studies are more likely to be published as journal editors prefer positive studies that show statistically significant differences to negative studies, and as a result, positive studies are found more often [53]. In this study, publication bias was evaluated using funnel plots and Egger’s regression test, and publication bias was confirmed in the results of the meta-analysis using data on fecal scores and survival rates. To overcome such errors, gray literature that has not been formally published should be used in a meta-analysis.

Since our study analyzed papers that were written and published in English and Korean, excluding research papers published in languages other than English and Korean from this analysis can be considered a limitation. In addition, using only the fecal scores and survival rates for the analysis as well as failing to analyze the virus output and antibody titers in the serum after challenge were other limitations.

To analyze the difference in the efficacy of PEDV vaccines based on vaccine type or vaccination route, vaccines were classified into killed vaccine and live vaccine and IM and PO, respectively, for analysis of their efficacy. In both cases, statistically significant differences were not found. Thus, differences in the efficacy of the vaccines based on vaccine type or vaccination route were not accepted. In some recent papers, it was reported that the efficacy of PEDV vaccines is questionable [8,9], but this is because cross-protection between different genotypes did not work due to the mutations in PEDV, and other researchers have already confirmed and reported that cross-protection of the PEDV G1a and G2b types partially works [23,29].

Immunization of pregnant sows is important for controlling the PED epidemic and reducing the number of deaths in suckling piglets [5,33]. Several live and killed PED vaccines that can be administered to sows are already commercially available on the market [9]. Our study suggests that the use of developed or commercially available PED vaccines could be a useful method of control and prevention of PED, regardless of the type of vaccine and route of administration.

## 5. Conclusions

A systematic review and meta-analysis were performed on studies that carried out a challenge test using virulent PEDV after vaccinating sows or piglets to confirm the efficacy of PEDV vaccines that were being developed or commercially sold. To evaluate the efficacy of the vaccines, data were analyzed based on the fecal scores and survival rates following the challenge test on the vaccinated and control groups. The results confirmed that there was a statistically significant effect size. Regarding differences in vaccine efficacy between vaccine types or vaccination routes, there was no statistically significant difference in the efficacy between the killed and live vaccines or between vaccination via intramuscular and oral administration.

## Figures and Tables

**Table 1:** Analysis of vaccines against porcine epidemic diarrhea based on published articles.

Virus Type	Route	Antigen Types		Target Animals	Clinical Observations in Piglets		Survival Rate	Detection of Antibody			Ref.
					Viral Shedding	Diarrhea		Neutralizing Antibody	ELISA		
									IgA	IgG	
Live	IM *	Attenuated virus		sow	N/A	Significantly improved	80.0% (*n* = 15)	Serum, milk	N/A	N/A	[23]
					N/A	Mild	68.0% (*n* = 25)	N/A	N/A	Serum, colostrum	[12]
	PO	Attenuated virus		sow	N/A	N/A	87.0% (*n* = 23)	Colostrum, whey	Colostrum	N/A	[13]
					Significantly reduced	Significantly reduced	100% (*n* = 30)	Serum, colostrum	Serum, colostrum	N/A	[24]
					N/A	No diarrhea	91.2% (*n* = N/A)	Serum, milk	Serum, milk	Serum, milk	[25]
					Mitigated	Mitigated	66.7% (*n* = 30)	Serum, colostrum	N/A	N/A	[26]
				piglet	Lower and delayed	None-to-mild	N/A	Serum	Feces	Serum	[27]
					Rapid declined	Significantly reduced	N/A	Serum	Serum	Serum	[28]
		Mutants virus	S-INDEL	piglet	Significantly lower	Reduced	N/A	Serum	Serum, feces	Serum	[29]
			2′-O-Mtase, endocytosis signal		Significantly lower	No diarrhea	100% (*n* = 5)	Serum	N/A	N/A	[30]
Killed	IN	whole virus		sow	Significantly lower	Lower diarrheal score	80.0% (*n* = 15)	Serum, colostrum	Colostrum	Serum, colostrum	[31]
					Significantly lower	Lower diarrheal score	86.7% (*n* = 15)	Serum, colostrum	Serum, colostrum	Serum, colostrum	[32]
	IM	whole virus		sow	Significantly reduced	Significantly reduced	91.7% (*n* = 24)	Serum	N/A	N/A	[33]
					Greatly reduced	Significantly reduced	100% (*n* = 5)	Serum, colostrum	Colostrum	Serum	[34]
				piglet	Reduced	No diarrhea	N/A	Serum	N/A	N/A	[17]
					Delayed	Mild	N/A	Serum	N/A	Serum	[35]
					Decreased	Mild	N/A	Serum	N/A	Serum	[36]
					N/A	Reduced	N/A	Serum	N/A	Serum	[37]
Killed	IM	recombinant	S1 protein	sow	Reduced	Mild	N/A	Serum, colostrum	N/A	N/A	[38]
			S1 protein		No significant differences	No significant differences	87.5% (*n* = 8)	Serum	Colostrum	Serum, colostrum	[39]
			S1 protein		Significantly reduced	Not mitigated	N/A	Serum, colostrum	Serum, colostrum	Serum, colostrum	[40]
			S protein		No significant differences	No significant differences	95% (*n* = 24)	Serum	Serum, colostrum	Serum, colostrum	[41]
			S protein		Reduced	Reduced	100% (*n* = 15)	Serum, colostrum	N/A	Serum	[42]
			S protein	piglet	Markedly decreased	Significantly reduced	N/A	Serum	Serum	Serum	[43]
			S protein		Not detected	No diarrhea	100% (*n* = 10)	Serum	Serum	Serum	[44]
			S protein		Significantly lower	Mild	N/A	Serum	Feces	Serum	[45]
			S protein		Reduced	Delayed	N/A	Serum	Feces	Serum	[46]
			S protein		Delayed	Delayed	N/A	Serum	Serum	Serum	[47]
			COE protein		Reduced	Lower diarrheal score	N/A	Serum	Serum, feces, saliva	Serum	[18]
	PO	recombinant	S1D protein on microsphere	sow	Lower copy numbers	Reduced	50.0% (*n* = 16)	Colostrum, whey	Colostrum, whey	N/A	[48]
			S protein in TGEV	piglet	Reduced	N/A	N/A	Serum	Serum	Serum	[49]
			COE protein in *L. casei*		Decreased	N/A	60.0% (*n* = 10)	N/A	Mucous extracts	Serum	[50]
			cDNA clone		Decreased	No diarrhea	N/A	Serum	Feces, saliva	Serum	[51]

* IM = intramuscularly; PO = per oral; IN = intranasally; N/A = not available

**Table 2:** The effect size analysis of the fixed effect and random effect model for porcine epidemic diarrhea virus (PEDV) vaccine efficacy. The effect size was converted into Hedges’ g for interpretation in this study, and is classified into small effect size (<0.3), medium effect size (0.3 to 0.8), and large effect size (≥0.8).

Outcome	Effect Model	Number	Effect Sizes	Standard Error	95% CI	*z*-Value
Fecal score	Fixed	12	1.907	0.162	1.589~2.224	11.775 *
	Random	12	2.421	0.402	1.634~3.208	6.028 *
Survival rate	Fixed	13	3.474	0.381	2.727~4.222	9.109 *
	Random	13	3.700	0.455	2.809~4.590	8.138 *

CI: confidence interval. * *p* < 0.05

**Table 3:** Heterogeneity test between the studies by fecal score and survival rate. The I^2^ statistic represents the degree of heterogeneity. Generally, heterogeneity is low for I^2^ values of ≤25%, moderate for up to 50%, and high for up to 75%. T^2^ represents the absolute value of the true variance (heterogeneity).

Outcome	Q-Value	df	*p*-Value	I^2^-Value	T^2^
Fecal score	55.586	11	0.000	80.211	1.374
Survival rate	15.286	12	0.226	21.498	0.552

df: degrees of freedom.

**Table 4:** Egger’s regression analysis for PEDV vaccine efficacy.

Item	Outcome	
	Fecal Score	Survival Rate
Intercept	3.044	2.446
Standard error	1.314	0.663
Lower 95% CI	0.114	0.986
Upper 95% CI	5.973	3.906
t-value	2.315	3.687
df	10	11
P (1-tailed)	0.002	0.001
P (2-taliled)	0.004	0.003

CI: confidence interval, df: degrees of freedom.

**Table 5:** The effect size analysis between the killed and live vaccines.

Outcome	Vaccine Type	Point Estimate	Standard Error	95% CI		Q-Value	df	*p*-Value
				Lower	Upper			
Fecal score	Killed	1.675	0.424	0.845	2.505	3.270	1	0.071
	Live	3.129	0.683	1.789	4.468			
Survival rate	Killed	4.109	0.672	2.792	5.426	1.317	1	0.251
	Live	3.173	0.463	2.264	4.081			

CI: confidence interval, df: degrees of freedom.

**Table 6:** The effect size analysis between the administration routes.

Outcome	Route	Point Estimate	Standard Error	95% CI		Q-Value	df	*p*-Value
				Lower	Upper			
Fecal score	IM *	2.043	0.250	1.063	3.023	0.880	1	0.348
	PO	2.849	0.489	1.479	4.220			
Survival rate	IM	3.383	0.466	2.470	4.297	0.116	1	0.734
	PO	3.659	0.664	2.358	4.600			

* IM: intramuscularly, PO: per oral, CI: confidence interval, df: degrees of freedom.

## References

[1] Kim S.H., Kim I.J., Pyo H.M., Tark D.S., Song J.Y., Hyun B.H. (2007). Multiplex real-time RT-PCR for the simultaneous detection and quantification of transmissible gastroenteritis virus and porcine epidemic diarrhea virus. J. Virol. Methods.

[2] Wood E.N. (1977). An apparently new syndrome of porcine epidemic diarrhoea. Vet. Rec..

[3] Pensaert M.B., De Bouck P. (1978). A new coronavirus-like particle associated with diarrhea in swine. Arch. Virol..

[4] Lee C. (2015). Porcine epidemic diarrhea virus: An emerging and re-emerging epizootic swine virus. Virol. J..

[5] Langel S.N., Paim F.C., Lager K.M., Vlasova A.N., Saif L.J. (2016). Lactogenic immunity and vaccines for porcine epidemic diarrhea virus (PEDV): Historical and current concepts. Virus Res..

[6] Cha T.-A., Kao K., Zhao J., Fast P.E., Mendelman P.M., Arvin A. (2000). Genotypic Stability of Cold-Adapted Influenza Virus Vaccine in an Efficacy Clinical Trial. J. Clin. Microbiol..

[7] Lee D.K., Park C.K., Kim S.H., Lee C. (2010). Heterogeneity in spike protein genes of porcine epidemic diarrhea viruses isolated in Korea. Virus Res..

[8] Song D., Huang D., Peng Q., Huang T., Chen Y., Zhang T., Nie X., He H., Wang P., Liu Q. (2015). Molecular characterization and phylogenetic analysis of porcine epidemic diarrhea viruses associated with outbreaks of severe diarrhea in piglets in Jiangxi, China 2013. PLoS ONE.

[9] Lv C., Xiao Y., Li X., Tian K. (2016). Porcine epidemic diarrhea virus: Current insights. Virus Adapt. Treat..

[10] Sato T., Takeyama N., Katsumata A., Tuchiya K., Kodama T., Kusanagi K. (2011). Mutations in the spike gene of porcine epidemic diarrhea virus associated with growth adaptation in vitro and attenuation of virulence in vivo. Virus Genes.

[11] Kweon C.-H., Kwon B.-J., Jung T.-S., Kee Y.-J., Hur D.-H., Hwang E.-K., Rhee J.-C., An S.-H. (1993). Isolation of porcine epidemic diarrhea virus (PEDV) in Korea. Korean J. Vet. Res..

[12] Kweon C.-H., Kwon B.-J., Lee J.-G., Kwon G.-O., Kang Y.-B. (1999). Derivation of attenuated porcine epidemic diarrhea virus (PEDV) as vaccine candidate. Vaccine.

[13] Song D.S., Oh J.S., Kang B.K., Yang J.S., Moon H.J., Yoo H.S., Jang Y.S., Park B.K. (2007). Oral efficacy of Vero cell attenuated porcine epidemic diarrhea virus DR13 strain. Res. Vet. Sci..

[14] Higgins J.P., Green S. (2008). Cochrane Handbook for Systematic Reviews of Interventions.

[15] Egger M., Smith G.D., Phillips A.N. (1997). Meta-analysis, Principles and procedures. BMJ.

[16] Noble J.H. (2006). Meta-analysis: Methods, strengths, weaknesses, and political uses. J. Lab. Clin. Med..

[17] Li Y., Wang G., Wang J., Man K., Yang Q. (2017). Cell attenuated porcine epidemic diarrhea virus strain Zhejiang08 provides effective immune protection attributed to dendritic cell stimulation. Vaccine.

[18] Li Q., Xu Z., Wu T., Peng O., Huang L., Zhang Y., Xue C., Wen Z., Zhou Q., Cao Y. (2018). A flagellin-adjuvanted PED subunit vaccine improved protective efficiency against PEDV variant challenge in pigs. Vaccine.

[19] Higgins J.P.T., Thompson S.G., Deeks J.J., Altman D.G. (2003). Measuring inconsistency in meta-analyses. BMJ.

[20] Egger M., Smith G.D., Schneider M., Minder C. (1997). Bias in meta-analysis detected by a simple, graphical test. BMJ.

[21] Lee J. (2008). Meta-analysis. Endocrinol. Metab..

[22] Duval S., Tweedie R. (2000). Trim and fill: A simple funnel-plot-based method of testing and adjusting for publication bias in meta-analysis. Biomertics.

[23] Sato T., Oroku K., Ohshima Y., Furuya Y., Sasakawa C. (2018). Efficacy of genogroup 1 based porcine epidemic diarrhea live vaccine against genogroup 2 field strain in Japan. Virol. J..

[24] Jang G., Won H., Lee D.U., Noh Y.H., Lee S.C., Choi H.W., Yoon I.J., Lee Y.J., Sang Yoo H., Lee C. (2019). Assessment of the safety and efficacy of an attenuated live vaccine based on highly virulent genotype 2b porcine epidemic diarrhea virus in nursing piglets. Vet. Microbiol..

[25] Wen Z., Xu Z., Zhou Q., Li W., Wu Y., Du Y., Chen L., Xue C., Cao Y. (2019). A heterologous ‘prime-boost’ anti-PEDV immunization for pregnant sows protects neonatal piglets through lactogenic immunity against PEDV. Lett. Appl. Microbiol..

[26] Won H., Lee D.U., Jang G., Noh Y.H., Lee S.C., Choi H.W., Yoon I.J., Yoo H.S., Lee C. (2019). Generation and protective efficacy of a cold-adapted attenuated genotype 2b porcine epidemic diarrhea virus. J. Vet. Sci..

[27] Chang Y.C., Kao C.F., Chang C.Y., Jeng C.R., Tsai P.S., Pang V.F., Chiou H.Y., Peng J.Y., Cheng I.C., Chang H.W. (2017). Evaluation and Comparison of the Pathogenicity and Host Immune Responses Induced by a G2b Taiwan Porcine Epidemic Diarrhea Virus (Strain Pintung 52) and Its Highly Cell-Culture Passaged Strain in Conventional 5-Week-Old Pigs. Viruses.

[28] Lin C.M., Ghimire S., Hou Y., Boley P., Langel S.N., Vlasova A.N., Saif L.J., Wang Q. (2019). Pathogenicity and immunogenicity of attenuated porcine epidemic diarrhea virus PC22A strain in conventional weaned pigs. BMC Vet. Res..

[29] Opriessnig T., Gerber P.F., Shen H., De Castro A., Zhang J., Chen Q., Halbur P. (2017). Evaluation of the efficacy of a commercial inactivated genogroup 2b-based porcine epidemic diarrhea virus (PEDV) vaccine and experimental live genogroup 1b exposure against 2b challenge. Vet. Res..

[30] Hou Y., Ke H., Kim J., Yoo D., Su Y., Boley P., Chepngeno J., Vlasova A.N., Saif L.J., Wang Q. (2019). Engineering a Live Attenuated Porcine Epidemic Diarrhea Virus Vaccine Candidate via Inactivation of the Viral 2′-O-Methyltransferase and the Endocytosis Signal of the Spike Protein. J. Virol..

[31] Li B., Du L., Yu Z., Sun B., Xu X., Fan B., Guo R., Yuan W., He K. (2017). Poly (d,l-lactide-co-glycolide) nanoparticle-entrapped vaccine induces a protective immune response against porcine epidemic diarrhea virus infection in piglets. Vaccine.

[32] Xu X., Du L., Fan B., Sun B., Zhou J., Guo R., Yu Z., Shi D., He K., Li B. (2020). A flagellin-adjuvanted inactivated porcine epidemic diarrhea virus (PEDV) vaccine provides enhanced immune protection against PEDV challenge in piglets. Arch. Virol..

[33] Baek P.S., Choi H.W., Lee S., Yoon I.J., Lee Y.J., Lee du S., Lee S., Lee C. (2016). Efficacy of an inactivated genotype 2b porcine epidemic diarrhea virus vaccine in neonatal piglets. Vet. Immunol. Immunopathol..

[34] Park J.E., Kang K.J., Ryu J.H., Park J.Y., Jang H., Sung D.J., Kang J.G., Shin H.J. (2018). Porcine epidemic diarrhea vaccine evaluation using a newly isolated strain from Korea. Vet. Microbiol..

[35] Lee S.H., Yang D.K., Kim H.H., Cho I.S. (2018). Efficacy of inactivated variant porcine epidemic diarrhea virus vaccines in growing pigs. Clin. Exp. Vaccine Res..

[36] Liu X., Zhang L., Zhang Q., Zhou P., Fang Y., Zhao D., Feng J., Li W., Zhang Y., Wang Y. (2019). Evaluation and comparison of immunogenicity and cross-protective efficacy of two inactivated cell culture-derived GIIa- and GIIb-genotype porcine epidemic diarrhea virus vaccines in suckling piglets. Vet. Microbiol..

[37] Liu X., Zhang Q., Zhang L., Zhou P., Yang J., Fang Y., Dong Z., Zhao D., Li W., Feng J. (2019). A newly isolated Chinese virulent genotype GIIb porcine epidemic diarrhea virus strain: Biological characteristics, pathogenicity and immune protective effects as an inactivated vaccine candidate. Virus Res..

[38] Oh J., Lee K.W., Choi H.W., Lee C. (2014). Immunogenicity and protective efficacy of recombinant S1 domain of the porcine epidemic diarrhea virus spike protein. Arch. Virol..

[39] Makadiya N., Brownlie R., Van den Hurk J., Berube N., Allan B., Gerdts V., Zakhartchouk A. (2016). S1 domain of the porcine epidemic diarrhea virus spike protein as a vaccine antigen. Virol. J..

[40] Subramaniam S., Yugo D.M., Heffron C.L., Rogers A.J., Sooryanarain H., LeRoith T., Overend C., Cao D., Meng X.J. (2018). Vaccination of sows with a dendritic cell-targeted porcine epidemic diarrhea virus S1 protein-based candidate vaccine reduced viral shedding but exacerbated gross pathological lesions in suckling neonatal piglets. J. Gen. Virol..

[41] Joshi L.R., Okda F.A., Singrey A., Maggioli M.F., Faccin T.C., Fernandes M.H.V., Hain K.S., Dee S., Bauermann F.V., Nelson E.A. (2018). Passive immunity to porcine epidemic diarrhea virus following immunization of pregnant gilts with a recombinant orf virus vector expressing the spike protein. Arch. Virol..

[42] Ke Y., Yu D., Zhang F., Gao J., Wang X., Fang X., Wang H., Sun T. (2019). Recombinant vesicular stomatitis virus expressing the spike protein of genotype 2b porcine epidemic diarrhea virus: A platform for vaccine development against emerging epidemic isolates. Virology.

[43] Hain K.S., Joshi L.R., Okda F., Nelson J., Singrey A., Lawson S., Martins M., Pillatzki A., Kutish G.F., Nelson E.A. (2016). Immunogenicity of a recombinant parapoxvirus expressing the spike protein of Porcine epidemic diarrhea virus. J. Gen. Virol..

[44] Yuan X., Lin H., Li B., He K., Fan H. (2017). Efficacy and immunogenicity of recombinant swinepox virus expressing the truncated S protein of a novel isolate of porcine epidemic diarrhea virus. Arch. Virol..

[45] Chang C.Y., Hsu W.T., Chao Y.C., Chang H.W. (2018). Display of Porcine Epidemic Diarrhea Virus Spike Protein on Baculovirus to Improve Immunogenicity and Protective Efficacy. Viruses.

[46] Chang Y.C., Chang C.Y., Tsai P.S., Chiou H.Y., Jeng C.R., Pang V.F., Chang H.W. (2018). Efficacy of heat-labile enterotoxin B subunit-adjuvanted parenteral porcine epidemic diarrhea virus trimeric spike subunit vaccine in piglets. Appl. Microbiol. Biotechnol..

[47] Liu X., Zhao D., Zhou P., Zhang Y., Wang Y. (2019). Evaluation of the Efficacy of a Recombinant Adenovirus Expressing the Spike Protein of Porcine Epidemic Diarrhea Virus in Pigs. Biomed. Res. Int..

[48] Choe S., Song S., Piao D., Park G.N., Shin J., Choi Y.J., Kang S.K., Cha R.M., Hyun B.H., Park B.K. (2020). Efficacy of orally administered porcine epidemic diarrhea vaccine-loaded hydroxypropyl methylcellulose phthalate microspheres and RANKL-secreting L. lactis. Vet. Microbiol..

[49] Pascual-Iglesias A., Sanchez C.M., Penzes Z., Sola I., Enjuanes L., Zuniga S. (2019). Recombinant Chimeric Transmissible Gastroenteritis Virus (TGEV)—Porcine Epidemic Diarrhea Virus (PEDV) Virus Provides Protection against Virulent PEDV. Viruses.

[50] Hou X., Jiang X., Jiang Y., Tang L., Xu Y., Qiao X., Min L., Wen C., Ma G., Li Y. (2018). Oral Immunization against PEDV with Recombinant Lactobacillus casei Expressing Dendritic Cell-Targeting Peptide Fusing COE Protein of PEDV in Piglets. Viruses.

[51] Kao C.F., Chiou H.Y., Chang Y.C., Hsueh C.S., Jeng C.R., Tsai P.S., Cheng I.C., Pang V.F., Chang H.W. (2018). The Characterization of Immunoprotection Induced by a cDNA Clone Derived from the Attenuated Taiwan Porcine Epidemic Diarrhea Virus Pintung 52 Strain. Viruses.

[52] Park S.-A., Cho M.-J. (2019). Effects of Exercise Intervention Programs on Recovery of Functional and Quality of Life in Breast Cancer Patients: A Systematic Review and Meta Analysis. Korean Parent Child. Health J..

[53] Simes R.J. (1987). Confronting publication bias: A cohort design for meta-analysis. Stat. Med..

